# Effect of *Camellia sinensis* teas on left ventricular hypertrophy and insulin resistance in dyslipidemic mice

**DOI:** 10.1590/1414-431X20209303

**Published:** 2020-04-27

**Authors:** M.C.L. Ferreira, L.N. Lima, L.H.T. Cota, M.B. Costa, P.M.E. Orsi, R.P. Espíndola, A.V. Albanez, B.B. Rosa, M.G.S. Carvalho, J.A.D. Garcia

**Affiliations:** 1 Faculdade de Medicina, Universidade José do Rosário Vellano, Alfenas, MG, Brasil; 2 Faculdade de Biomedicina, Universidade José do Rosário Vellano, Alfenas, MG, Brasil; 3 Faculdade de Educação Fisíca, Universidade José do Rosário Vellano, Alfenas, MG, Brasil; 4 Curso de Ciências Biológicas, Instituto Federal do Sul de Minas, Machado, MG, Brasil; 5 Programa de Pós-Graduação em Reprodução Animal, Sanidade e Bem Estar Animal, Universidade José do Rosário Vellano, Alfenas, MG, Brasil

**Keywords:** Lipids, Cardiovascular system, Tea, Glucose, Insulin

## Abstract

The control of dyslipidemia using plants is an important subject of studies since it has numerous benefits in cardiovascular protection. The objective of this study was to evaluate the effect of three *Camellia sinensis* L. teas (green, red, and white) on left ventricular hypertrophy and insulin resistance in low-density lipoprotein receptor knockout (LDLr-/-) mice fed a high-fat diet. The LDLr-/- mice were divided into four experimental groups: Group C: standard feed; Group CT: standard feed and three teas, Group HL: high-fat feed; HLT Group: high-fat feed and three teas. The three types of tea (green, red, and white) originated from different processing of the *Camellia sinensis* L. plant, and were administered associated once a day at a dose of 25 mg/kg by gavage for 60 days. The teas partially prevented hyperlipidemia, the decrease of the serum levels of high-density lipoproteins (HDL), insulin resistance, and increased C-reactive protein (CRP) levels, and completely prevented left ventricular hypertrophy in LDLr -/- mice of the HLT group. In conclusion, the three *Camellia sinensis* L. teas used to control genetic dyslipidemia associated with a high-fat diet can be used as an auxiliary treatment associated with the control of lipid intake, thus promoting cardiac protection against hyperlipidemia.

## Introduction

Dyslipidemias are changes in the concentrations of circulating lipids originating from metabolic processes and diets and are classified as isolated hyperlipidemias (hypercholesterolemia or hypertriglyceridemia), mixed hyperlipidemia (increase in the serum concentrations of total cholesterol (TC) and triglycerides (TG)), and hypolipidemia (decrease in the serum concentrations of high-density lipoproteins (HDL)) (1,2). The conditions that promote and sustain cardiac hypertrophy in knockout mice for the low-density lipoprotein receptor gene (LDLr-/-) fed high-fat diets are the inflammatory process via CD40L (3), oxidative stress, and insulin resistance (4) resulting from dyslipidemia. Left ventricular hypertrophy (LVH) is a highly relevant indicator of the risk of cardiovascular morbidity and mortality (5).

Treating dyslipidemia is one of the best forms to prevent comorbidities such as LVH and atherosclerosis. Thus, the non-pharmacological treatment of dyslipidemia is based on the orientation of a healthy, low-fat, and low-calorie diet, combined with physical activity (2). However, the use of drugs to treat dyslipidemia and diseases of which pathogenesis is related to dyslipidemia, such as coronary artery disease, cerebrovascular disease, and peripheral vascular disease, is often recommended (2,6). The most commonly used medications are statins from the class of 3-hydroxy-3-methylglutaryl coenzyme A (HMG CoA) reductase inhibitors (6). These medications present side effects such as myopathy, the most common side effect that can appear within weeks or years after the beginning of treatment. Myopathy has a wide clinical spectrum, ranging from myalgia, with or without the increase of creatine kinase, to rhabdomyolysis, which can be fatal (2,6).

Alternative treatments presenting fewer side effects, such as herbal medicines, have been widely reported (7). The use of herbal medicines has increased worldwide, with an annual estimate of 10 to 20% of the commercial movement of the pharmaceutical market (8). *Camellia sinensis* teas, derived from the processing of different plants such as red, green, white, and black, are considered one of the most consumed beverages in the world (7,9) and can be considered an herbal medicine for dyslipidemia and its consequent pathologies. The increasing popularity of herbal medicines is due to many factors including the belief that natural products are free of toxins and effective for treating new and old diseases that have unsatisfactory conventional treatments (8). Scientifically, *Camellia sinensis* teas have shown antioxidant and anti-inflammatory effects, given that they are rich in catechins, theobromine, flavonoids, polyphenols, caffeine, and micronutrients such as vitamins B, E, and C, and minerals, such as calcium, magnesium, zinc, potassium, and iron (9,10). In addition to herbal medicines, functional foods are also considered alternative treatments (9). Thus, the objective of this study was to evaluate the effect of three associated teas (tri-tea), green, red, and white, derived from *Camellia sinensis* on the prevention of left ventricular hypertrophy and insulin resistance in knockout mice for the LDLr gene fed a high-fat diet.

## Material and Methods

### Animal protocol

The experiments were conducted using 40 male mice, homozygote for the LDL receptor gene (LDLr-/-), with three months of age and weighing 22±3 g. The animals were maintained under controlled temperature (22±3°C) and 12-h light/dark cycle.

The mice were divided into four groups of ten mice each. All animals received water and feed *ad libitum* for 60 days. Group C received the standard feed, Group CT received standard feed and tri-tea, Group HL received the high-fat feed, and Group HLT received high-fat feed and tri-tea. The standard feed for rodents contains 4% total fat, while the high-fat feed contains 20% total fat with 1.25% cholesterol and 0.5% cholic acid. The three associated teas (green, red, and white) originated from different processing treatments of the *Camellia sinensis* plant, and were administered once daily at a dose of 25 mg/kg through gavage for 60 days. The groups that did not receive tea received water via gavage to simulate the manipulation.

After 60 days of experimentation, the mice were fasted for 8 h followed by local intraperitoneal anesthesia using 6 mg/kg xylazine and 40 mg/kg ketamine (Bayer AS and Parke-Davis^®^, USA). Blood samples were collected by retro-orbital venous plexus puncture to analyze the serum concentrations of TG, TC, low-density lipoprotein (LDL), and HDL. After euthanasia by deepening the anesthesia with sodium thiopental at a dose of 100 mg/kg, applied intraperitoneally, a thoracotomy was performed, surgically removing the heart. After the experiment, the animals were frozen and incinerated. The experimental procedures were conducted according to directives established by the Concelho Nacional de Controle de Experiencias Animal (CONCEA: National Council for Animal Experiment Control) and approved by the Animal Ethics Committee of the Universidade José do Rosário Vellano (UNIFENAS, Brazil; No. 10A/2016).

### Serum analysis

Serum was obtained by centrifuging the blood (1200 *g*, 4°C, 10 min). TG, TC, and high-density lipoprotein cholesterol (HDLc) were measured using the colorimetric enzymatic methods described by Hedrick et al. (11). The level of CRP was determined by turbidimetry and photometry (Humastar 300SR, Human Diagnostics, Germany). Glucose was measured by the colorimetric enzymatic method. Insulin was determined using an ELISA commercial kit (DAKO Ltd., UK). The Homa index (Homa-ir) was calculated using the formula: {Homa-ir = [fast insulinemia (mU/L) × fast glycemia (mM)] / 22.5} to determine resistance to insulin.

### Histological procedures

Soon after its removal, the heart was dissected and the left ventricle isolated. To determine left ventricular hypertrophy, the left ventricular weight (mg) to mouse body weight (g) ratio was calculated. The left ventricles were fixed for 24 h in 10% formalin. Subsequently, they were embedded in paraffin to obtain histological sections at a thickness of four micrometers (12).

The histological sections of the ventricles were stained with hematoxylin/eosin for cardiomyocyte morphometric analysis. Twelve transverse histological sections of each ventricle were performed. Then, eight photomicrographs (400×) of each section were obtained from the same pre-fixed point using a digital camera coupled to the Leica IM50 software program (version 1.20, Germany). The diameters of 8 to 12 cardiomyocytes of each photomicrograph (13) were measured, calculating the mean diameter of the cardiomyocytes of each animal and the average diameter of the cardiomyocytes per group.

Other histological sections of the ventricles were stained with picrosirius red to evaluate and quantify cardiac tissue collagen and were analyzed with polarized light. Each photomicrograph was analyzed using the SIACS (System for Image Analysis of Cells Structures, Brazil; Registration number: BR512015000558-8) obtaining the fractional percentages of collagen from the areas marked in red (14).

All histological analyses were performed by a single examiner using the double-blind method.

### Statistical analysis

The number of animals required was determined based on statistical planning and the blood volume necessary because the animals are small. Data are reported as means±SE. ANOVA followed by the Tukey's test was used to compare the means between groups. The differences were considered significant when P<0.05.

The data were organized using Excel^®^ software from the Office 2016 package (Microsoft, USA). Subsequently, the data were processed using the GraphPad Prism 8.0^©^ software (USA).

## Results

Evaluation of the lipid profile showed that the *Camellia sinensis* tri-tea prevented the decrease of serum levels of HDL in HLT mice compared with HL mice. Furthermore, the teas partially prevented the increase in the serum concentrations of TG and TC in HLT mice compared with HL mice. Moreover, the tri-tea presented no effect on the lipid profile of CT mice and showed no differences compared with mice from Group C (Table 1). There was no difference between the glycemic profiles of the studied groups. However, the serum levels of insulin increased in the HL group, and tri-tea partially prevented the increase in serum insulin in the HLT mice, improving insulin resistance. The tri-tea also prevented the increase of serum levels of CRP in the HLT group.

**Table 1:** Serum levels of total cholesterol (TC), triglycerides (TG), high-density lipoprotein cholesterol (HDLc), glucose, insulin, C reactive protein (CRP), and left ventricle weight (mg)/mouse weight (g) ratio, cardiomyocyte diameter, and interstitial collagen area in the left myocardium of LDLr-/- mice fed a standard diet (C), standard diet with tri-tea (CT), high-fat diet (HL), or high-fat diet with tri-tea (HLT).

	C (n=9)	CT (n=7)	HL (n=8)	HLT (n=7)
TC (mg/dL)	254±8^c^	246±4^c^	758±17^a^	432±11^b^
TG (mg/dL)	140±5^c^	135±6^c^	248±12 ^a^	186±8^b^
HDLc (mg/dL)	62±5^c^	58±3^a^	28±4^c^	46±6^b^
Glucose (mM)	5.7±0.4	5.2±0.6	5.9±0.8	5.8±0.6
Insulin (mU/L)	2.3±0.5^c^	2±0.5^c^	6.5±1^a^	4.1±0.4^b^
Homa-ir	0.6±0.08^c^	0.5±0.06^c^	1.7±0.1^a^	1±0.09^b^
CRP (mg/dL)	6.2±0.5^c^	5.8±0.8^c^	13±2^a^	8.2±1^b^
Left ventricle weight (mg)/mouse weight (g) ratio	3.3±0.06^b^	3.3±0.10^b^	4.0±0.10^a^	3.4±0.14^b^
Cardiomyocyte diameter (μm)	18±1.2^b^	18±1.9^b^	24.6±1.5^a^	20±1.8^b^
Interstitial collagen area in the left myocardium (%)	3.2±0.2^c^	3.5±0.3^c^	9.9±0.8^a^	5.7±0.5^b^

Data are reported as means±SE. Means followed by the same letter on lines do not differ significantly (P<0.05, Tukey's test).

**Figure 1:**
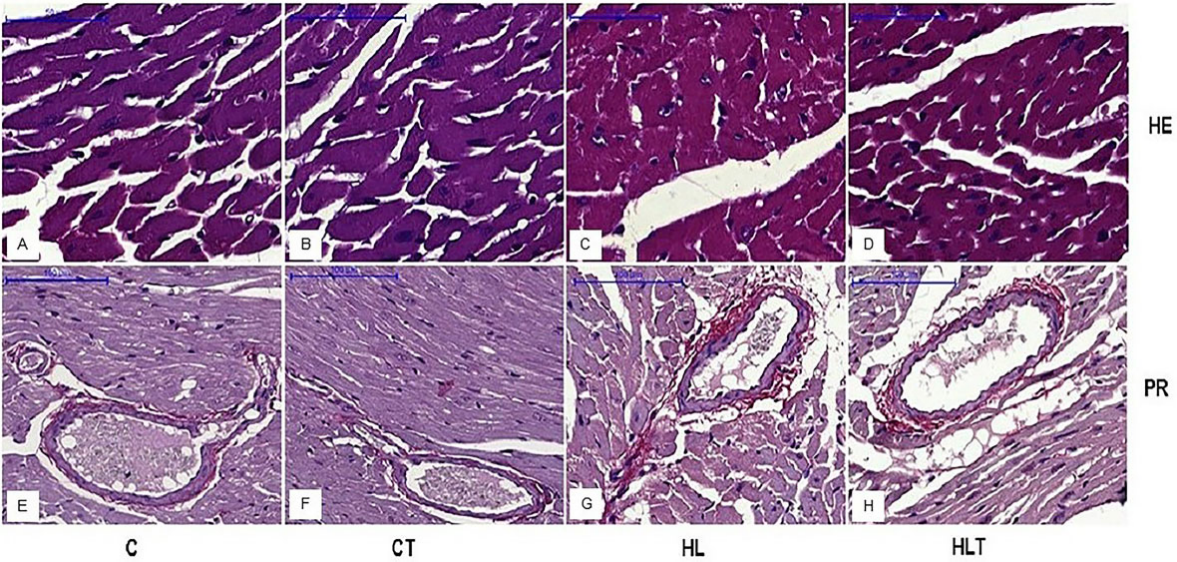
**A**–**D**, Photomicrographs of the left ventricle, showing the diameter of the cardiomyocytes, stained with hematoxylin and eosin (HE). **E**–**H**, Photomicrographs showing the distribution of interstitial and perivascular staining with red collagen (marked red by the dye) in the left ventricular myocardium of picrosirius (PR) stained mice. Groups: C, standard diet; CT, standard diet with tri-tea; LH, high-fat diet; HLT, high-fat diet with tri-tea. Scale bar: 100 μm.

There was an increase in the left ventricular weight (mg)/mouse weight (g) ratio in the animals from the HL group compared with those of Group C. The LVH of HL mice was correlated with an increase in the diameter of the cardiomyocytes and deposition of interstitial and perivascular collagen. The tri-tea prevented LVH in HLT mice, completely inhibiting the increase in the diameter of the cardiomyocytes and partially hindering the deposition of interstitial and perivascular collagen. The tri-tea had no effect on the cardiac histological parameters evaluated in the mice from the CT group compared to those of the C group (Table 1 and Figure 1).

## Discussion

Previous studies conducted in our laboratory have demonstrated that LDLR-/- mice fed high-fat diets developed severe mixed hyperlipidemia followed by a decrease in the serum levels of HDL and left ventricular hypertrophy (4,15–17), which involves CD40L signaling (3). Furthermore, they presented lipid peroxidation and protein oxidation associated with insulin resistance with hyperinsulinemia (4,15) and increased serum levels of CRP (4). In the present study, the associated teas (green, red, and white) from *Camellia sinensis* partially prevented hyperlipidemia, reduced serum levels of HDL, insulin resistance, and increased CRP levels, while completely preventing LVH in LDLR-/- mice fed a high-fat diet.

Studies have shown that catechins from *Camellia sinensis* reduced the activity of HMG-CoA reductase by competing with cholesterol at their binding sites (18), decreasing cholesterol production, the digestion of gastric and intestinal fat mediated by the direct inhibition of gastric and pancreatic lipases, and the lipid emulsification process (19), consequently reducing the intestinal absorption of lipids. Moreover, the catechins, theobromine, flavonoids, and polyphenols present in *Camellia sinensis* increased the plasmatic purification of lipids (10) and the intestinal peristaltic movements (20), increasing lipid excretion through feces. These studies showed several mechanisms involved with the hypolipidemic effect of *Camellia sinensis* teas, which could explain the partial prevention of mixed hyperlipidemia in the LDLr-/- mice of the HLT group that concomitantly received a high-fat diet and green, red, and white teas.

The mice from the HL group presented LVH similar to that developed in our laboratory, which demonstrated that LDLr-/- mice fed a high-fat diet developed LVH and atherosclerosis due to the inflammatory process initiated by severe dyslipidemia, associated with the decrease of the bioavailability of nitric oxide, in the serum levels of HDL, and of its antioxidant and anti-inflammatory functions (3,15,17,21,22). Furthermore, LDLr-/- mice fed a high-fat diet showed insulin resistance, with hyperinsulinemia associated with increased lipid peroxidation and protein oxidation (4). The *Camellia sinensis* teas prevented LVH, and partially prevented insulin resistance and increased levels of CRP. The lower oxidative stress prevented the hepatic removal of HDL with a consequent increase in serum HDL, an endogenous antioxidant and anti-inflammatory (23), and may have increased the bioavailability of nitric oxide, which has cardiac anti-hypertrophic properties (24), hindering the development of LVH. Therefore, a decrease in the concentrations of total cholesterol and triglycerides, associated with a decrease in insulin and an increase in the serum levels of HDL, contributed to the prevention of LVH in HLT mice.

The polyphenols from *Camellia sinensis* teas, most of which are flavonoids, present anti-inflammatory and antioxidant properties to various biological systems (25). Polyphenolic compounds are well known to be responsible for the antioxidant properties of many plants (26,27). The epigallocatechin-3-gallate catechin (a flavonoid) has played a key role as an antioxidant in the prevention and treatment of many diseases (25,28,29). Furthermore, *Camellia sinensis* teas have other flavonoids (such as quercetin, kaempferol, myricetin), phenolic acids (gallic and chlorogenic acids), proanthocyanidins (prodelphinidine), xanthic bases (caffeine, theophylline), polysaccharides, and essential amino acids (such as glycine, serine, valine, leucine, threonine, and characteristic amino acid theanine) (28,30). Although the mechanism is not entirely clear, *Camellia sinensis* had a beneficial effect on the cardiovascular system of the dyslipidemic mice used in this study. Polyphenols can be direct antioxidants, capturing reactive oxygen species or chelating transition metals (25,31). Alternatively, they may act indirectly by increasing the regulation of phase II antioxidant enzyme, which may have contributed to reducing oxidative stress markers in the HLT group, partially preventing insulin resistance and completely preventing LVH generated from severe mixed hyperlipidemia of the LDLr-/- mice fed a high-fat diet.

However, it is important to emphasize that *Camellia sinensis* teas, especially green tea, can present adverse effects (9). People with heart problems, especially arrhythmias, must avoid drinking too much green tea because of its action on the cardiovascular and respiratory systems. The excessive use of *Camellia sinensis* causes intoxication, characterized by tachycardia, nervous system excitement, seizures, delusions, and headache (32).

Despite the expanding use of medicinal plants, phytotherapy, and the relevant results such as those cited in the specific literature and suggested in this study, the subject is often poorly explored by the media and commerce (32). Further scientific research is needed for the safe use of these functional plants since their benefits are sometimes overlooked and may be alternatives to the use of drugs with severe side effects, such as rhabdomyolysis, caused by statins which are currently the most commonly used to treat dyslipidemia. Thus, further studies on the possible side effects of *Camellia sinensis* teas are necessary to inhibit its indiscriminate use.

In conclusion, the *Camellia sinensi*s teas used to control genetic dyslipidemia associated with a high-fat diet could be used as auxiliary treatment together with the control of fat intake. The *Camellia sinensis* teas completely prevented LVH, with partial effects on the lipid profile and prevention of insulin resistance of LDLR-/- mice fed high-fat diets.
